# Survival of rats bearing advanced intracerebral F 98 tumors after glutathione depletion and microbeam radiation therapy: conclusions from a pilot project

**DOI:** 10.1186/s13014-018-1038-6

**Published:** 2018-05-10

**Authors:** E. Schültke, E. Bräuer-Krisch, H. Blattmann, H. Requardt, J. A. Laissue, G. Hildebrandt

**Affiliations:** 1 Department of Radiooncology, Rostock University Medical Center, Südring 75, 18059 Rostock, Germany; 2 European Synchrotron Radiation Facility (ESRF), Grenoble, France; 3 Untersiggenthal, Switzerland; 4 Institute of Anatomy, University of Bern, Bern, Switzerland

**Keywords:** Animal model, Malignant brain tumour, Microbeam radiation therapy, Radioenhancement, Synchrotron X-rays

## Abstract

**Background:**

Resistance to radiotherapy is frequently encountered in patients with glioblastoma multiforme. It is caused at least partially by the high glutathione content in the tumour tissue. Therefore, the administration of the glutathione synthesis inhibitor Buthionine-SR-Sulfoximine (BSO) should increase survival time.

**Methods:**

BSO was tested in combination with an experimental synchrotron-based treatment, microbeam radiation therapy (MRT), characterized by spatially and periodically alternating microscopic dose distribution. One hundred thousand F98 glioma cells were injected into the right cerebral hemisphere of adult male Fischer rats to generate an orthotopic small animal model of a highly malignant brain tumour in a very advanced stage. Therapy was scheduled for day 13 after tumour cell implantation. At this time, 12.5% of the animals had already died from their disease.

The surviving 24 tumour-bearing animals were randomly distributed in three experimental groups: subjected to MRT alone (Group A), to MRT plus BSO (Group B) and tumour-bearing untreated controls (Group C). Thus, half of the irradiated animals received an injection of 100 μM BSO into the tumour two hours before radiotherapy.

Additional tumour-free animals, mirroring the treatment of the tumour-bearing animals, were included in the experiment. MRT was administered in bi-directional mode with arrays of quasi-parallel beams crossing at the tumour location. The width of the microbeams was ≈28 μm with a center-to-center distance of ≈400 μm, a peak dose of 350 Gy, and a valley dose of 9 Gy in the normal tissue and 18 Gy at the tumour location; thus, the peak to valley dose ratio (PVDR) was 31.

**Results:**

After tumour-cell implantation, otherwise untreated rats had a mean survival time of 15 days. Twenty days after implantation, 62.5% of the animals receiving MRT alone (group A) and 75% of the rats given MRT + BSO (group B) were still alive. Thirty days after implantation, survival was 12.5% in Group A and 62.5% in Group B. There were no survivors on or beyond day 35 in Group A, but 25% were still alive in Group B. Thus, rats which underwent MRT with adjuvant BSO injection experienced the largest survival gain.

**Conclusions:**

In this pilot project using an orthotopic small animal model of advanced malignant brain tumour, the injection of the glutathione inhibitor BSO with MRT significantly increased mean survival time.

## Background

Glioblastoma multiforme (WHO IV) is a highly malignant brain tumour listed as a rare disease [1], with a reported incidence of 2–3/100,000 per year in the USA and most European countries [2]. This equals about 2000 to 3000 new cases annually for a larger country like Germany and several hundred for a small country like Switzerland. The median survival time after diagnosis is about 1–2 years [3].

Metastudies show that radiotherapy is the only independent factor determining outcome in glioblastoma multiforme [4, 5]. One reason for the low success rate of current radiotherapy protocols has been attributed to the low radiosensitivity of glioblastoma multiforme [6], the extent of radioresistance perhaps directly correlated with patient outcome [7]. This radioresistance may be caused, at least in part, by the high glutathione content of the tumour [8, 9]. Glutathione enables the cells to quench a large percentage of the free radicals generated by radiotherapy, thereby acting as cytoprotective agent [10]. Oxidative damage to lipid membranes and subsequent functional loss can thus be limited. Therefore, we hypothesized that administration of the glutathione synthesis inhibitor Buthionine-SR-Sulfoximine (BSO) as adjuvant with radiotherapy should increase survival time. Intra-peritoneal administration of this synthetic amino acid analogue significantly reduced the cellular glutathione content in human glioma xenografts, although overall survival times were not prolonged [11, 12]. Conversely, in animal models of malignant brain tumours, BSO administration has increased survival times when given as adjuvant with chemotherapy [13] combined with Iodine 125 seeds [14], or when BSO was combined with conventional radiotherapy [15].

We have now combined the administration of the glutathione synthesis inhibitor BSO with a new experimental radiotherapeutic paradigm, in a small animal model of a very aggressive malignant brain tumour in an advanced stage of development. This paradigm, proposed for the treatment of malignant neoplasms, is a unique micro-radiosurgical method based on the principles of grid radiotherapy using synchrotrons X-rays [16, 17]. If a suitable collimator is inserted in the X-ray beam generated by a synchrotron, arrays of quasi-parallel microbeams with individual beam widths of up to 100 μm can be generated [18, 19]. This novel spatially and periodically alternating dose distribution at the microscopic level is the hallmark of microbeam radiation therapy (MRT). The tolerance of the normal brain tissue for MRT appears to be exceptionally high. This has been shown particularly with regard to acute radiation-induced damage such as edema and necrosis [20], even in the still developing brain of young animals [21–24]. X-ray doses up to two orders of magnitude higher than those ordinarily used in clinical radiooncology can be administered in one single fraction of MRT without causing white matter necrosis, thus taking the idea of spatial hypofractionation to an extreme. Experimental data suggest that tumour control with MRT might be superior to the control that can be achieved with comparable broad beam doses, even when administered in a single fraction [21, 22, 25–27]. From a clinical aspect it is important to note that MRT administered in therapeutically suitable doses does not appear to result in a significant impairment of normal behaviour. This has been shown in animal models of weanling piglets up to 2 years post irradiation [28] and in adult rats [27].

In the clinical radiotherapy of the brain, dose limits are dictated by the risks of cerebral edema, brain tissue necrosis and longterm changes in the white matter which can lead to cognitive deficits. MRT might be a good approach to overcome those limitations.

## Methods

Tumour cell implantation and irradiation with the aim to study the potential of the glutathione inhibitor BSO in combination with MRT in vivo were conducted at ID 17, the biomedical beamline of the European Synchrotron Radiation Facility (ESRF) in Grenoble, France.

### Animal model and group distribution

**Fig. 1:**
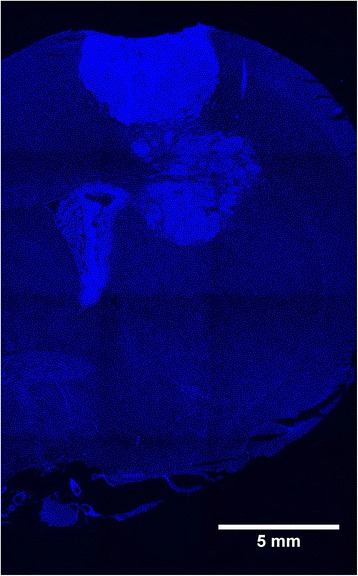
Histology of the right cerebral hemisphere, adult rat brain. DAPI stain for nuclei, paraffin section. Increased cellular density is one of the hallmarks of a highly malignant tumour. Tiled image after software-based stitching. Like the human glioblastoma multiforme at an advanced stage, the small animal model is that of a large space-occupying multifocal lesion

**Fig. 2:**
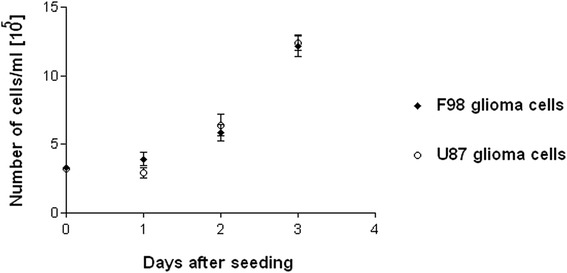
The proliferation pattern of human U87 glioma cells and F98 glioma cells (rat) is very similar in vitro. Two counts were made of triplicate cell cultures at the day of seeding and on days 1, 2 and 3 after seeding. 10 μl of the harvested cell suspension were diluted 1:9 in Trypan Blue, mixed thoroughly; 10 μl of this mixture were injected into each of the two counting chambers of the haematocytometer (Neubauer chamber). Cells were counted in four 4 × 4 small square areas, the four results were added and the resulting sum was divided by four

F98 glioma cells from a commercially available cell line (CRL-2397, ATCC) were used to generate orthotopic brain tumours in 27 male young adult Fischer rats (275–305 g). F98 gliomas share many characteristics with the cells of the highly malignant human brain tumour glioblastoma multiforme, such as fast aggressive growth with infiltration of normal brain structures (Fig. 1) and development of necrotic areas [29]. In our cell culture work we have noticed that, in vitro, the proliferation patterns of the human glioblastoma cell line U87 (ATTC HTB-14) and the rodent-derived F98 cell line are very similar, with doubling times around 24 h within the first three days after seeding (Fig. 2). This observation is in accordance with published data [30]. Furthermore, F98 gliomas are fairly radioresistant [29, 31]. F98 gliomas have been described as only weakly immunogenic, tumour masses rapidly increasing in size, characterized by multifocal necroses and parenchymal infiltration at the tumour margins [32]. Therefore, we consider the F98 glioma to be a suitable model for experimental radiotherapy studies aiming to develop a new therapeutic approach for the treatment of patients with highly malignant brain tumours.

The animals were housed and cared for in a temperature-regulated animal facility exposed to a 12-h light/dark cycle.

Ten million F98 glioma cells were harvested from cultures, suspended in 1 ml of Hanks serum and stored on ice. For each animal, 10 μl of this cell suspension were aspirated in a Hamilton syringe which was mounted on the vertical arm of a small animal stereotactic frame. Under general anaesthesia (1.5–2% Isoflurane^R^ in air inhalation), the scalp of the animals was shaved and disinfected. A sagittal midline incision was made in the scalp. The periosteum was retracted. A burr hole was placed 3 mm to the right of the sagittal suture and 3 mm posterior to the coronal suture. Using a small animal stereotactic frame, one hundred thousand F98 glioma cells were implanted into the anterior aspect of the right cerebral hemisphere of 27 rats. The cell suspension was injected through a 27 G needle attached to the Hamilton syringe, the tip of which was carefully lowered through the burr hole and inserted 3 mm below the cortical surface.

The cell suspension was injected over a period of 4 min (2.5 μl / min) using an automated injector pump (KDS310, Geneq, Montreal, Canada) to limit injury to the brain from the injection process. After completion of the tumour cell injection, the needle was left in place for an additional minute to allow the cell suspension to evenly distribute within the tissue. The needle was then withdrawn, the burr hole sealed with bone wax and the scalp sutured. The animals were allowed to recover. For analgesia, each animal received one dose of 0.05 mg Buprenorphine^®^/kg s.c. before surgery and a second dose at 12 h after surgery.

**Fig. 3:**
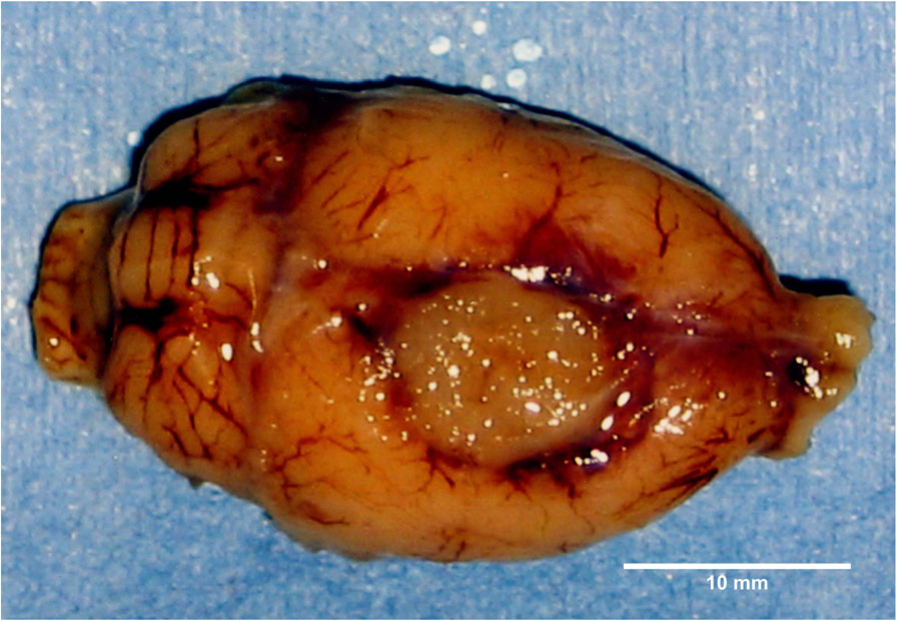
Dissected rat brain with tumour (about 10 mm ap × 8 mm lr) 3 days. After implantation of 100,000 F98 glioma cells in the right hemisphere. Note the deviation of the midline structure towards the left hemisphere. The animal died a few hours before the scheduled irradiation

Out of our 27 tumour-bearing animals, three animals (12.5%) died before the day of the scheduled radiotherapy from their progressive disease (Fig. 3). These animals underwent necropsy to assure that death occurred indeed due to tumour progression after injection into the proper location, and not due to potentially lethal haemorrhage.

### Microbeam radiation therapy (MRT)

The white X-ray beam generated by the synchrotron source and the wiggler was filtered (1.42 mm of C, 3.15 mm of Al and 1.75 mm of Cu), resulting in a spectrum that extends from about 50 to well above 350 keV, with a mean energy of 105 keV [33]. The microbeam array was generated using the TECOMET^R^ collimator with slits spaced 400 μm in the instrument. Because of the minimal divergence of the beam, the spacing was 411 μm at the level of the goniometer [19].

**Fig. 4:**
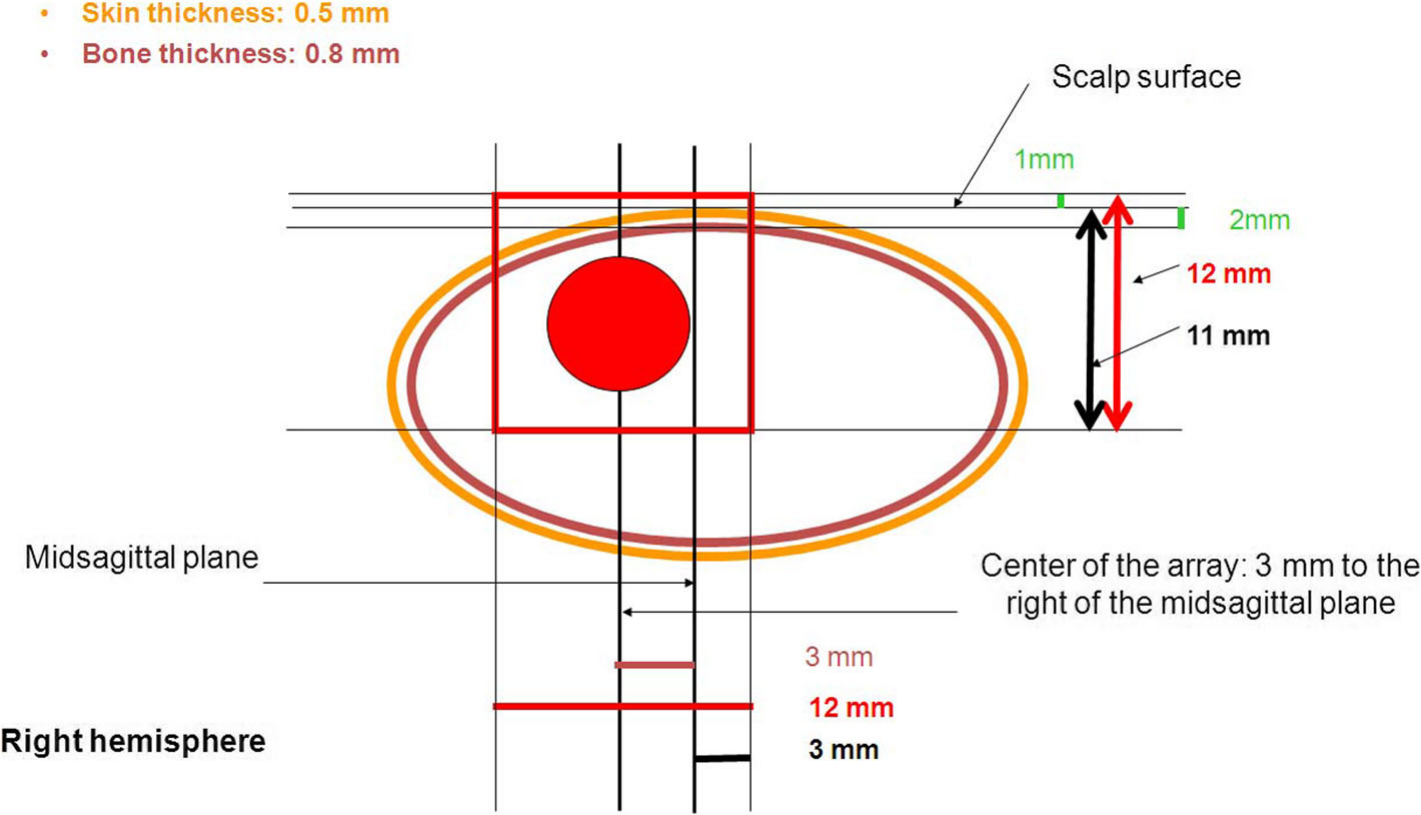
Schematic size and position of the projected lateral irradiation field (12 mm × 12 mm) with respect to tumour size and location

The tumours were expected to have diameters of 4.5–6.0 mm at irradiation on day 13 after tumour cell implantation. In the absence of an option for pre-therapeutic imaging, we irradiated a large tissue volume surrounding the tumour using two crossfired arrays centered on the tumour, each array ≈10 mm wide and 14 mm high, comprising 50 microplanar parallel microbeams of ≈28.3 μm FWHM at the level of the goniometer, with a center-to-center distance of ≈205.5 μm between adjacent microbeams (Fig. 4). While the variance in tumour size might be a limitation from the statistical point of view, it certainly represents the variability in tumour size seen in human patients with glioblastoma multiforme. Statistical power should be strengthened by increasing the number of animals per group.

The animals were irradiated by moving them vertically through the ≈0.5 mm high beam twice, with a horizontal movement between the two scans to halve the spacing distance from 411 μm to 205.5 μm.

The dose rate was measured for broad beam conditions in solid water plates (Goettingen White Water; 30 × 30 × 12 cm^3^) [34] using a Pinpoint ionization chamber (PTW, Ref. 31014). The chamber was calibrated with TH200 beam quality using an X-ray generator at mean energy of 109 keV, which is very close the MRT filtered spectrum resulting in a mean energy of 105 keV [33]. With the help of the MRT Graphical User Interface (GUI) the measured dose rate under reference conditions was entered and an adequate speed for the vertical translation was calculated, taking into account the machine current in the storage ring including Monte Carlo pre-calculated output factors for the microbeam width. The 350 Gy peak entrance dose at 3 mm depth resulted in approximately 280 Gy at the centre of the tumour.

According to our Monte Carlo calculations, the value of the peak-to-valley dose ratio (PVDR) for MRT with the parameters used in our study was around 31. Therefore, the MRT valley dose would have been about 9 Gy per port for the 350 Gy peak entrance dose. Thus, in the normal tissue, the valley dose in our experimental design would have agreed with the recommendations resulting from the QUANTEC study, which was aimed at determining threshold values for normal tissue tolerance [35]. Only at the tumour location (crossed beam arrays), the valley dose was 18 Gy.

The surviving 24 tumour-bearing animals were randomly distributed into three experimental groups (*n* = 8 per group): Animals in Group A were submitted to MRT alone. Animals in Group B also underwent MRT but additionally received one injection of BSO into their tumours two hours before MRT. Using the small animal stereotactic frame again, four microliter of a 100 μM BSO solution were injected through the burr hole which had been used previously for tumour cell implantation, with the tip of the needle 3 mm below the cortical surface. Thus, BSO was injected in the same location where the tumour cells had been deployed 13 days earlier and the BSO was assumed to be deposited in the centre of the tumour that had developed from those F98 glioma cells. The dose of BSO was chosen based on the results of experiments described by Ataelmannan [15]. The animals in Group C served as tumour-bearing untreated controls.

Irradiation of the animals was conducted under general anaesthesia (chloral hydrate, 0.4 g / kg rat).

**Fig. 5:**
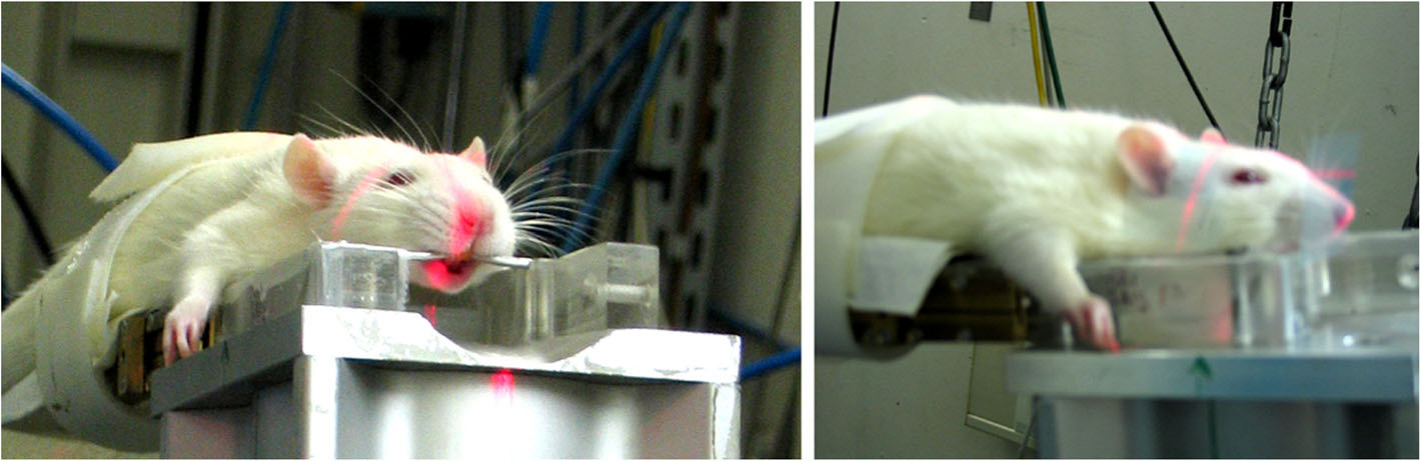
Positioning for microbeam irradiation. Laser beams mark the center of the irradiation field. The laser lines (red) mark the centre of the anterior-posterior (left) and lateral (right) microbeam arrays, crossing at the tumour location. Note that there is a 3 mm offset towards the right of the midsagittal line

MRT was administered in a single session, with a peak skin entry dose of 350 Gy in each direction. The rats were positioned in a prone position on the goniometer, orthogonally to the direction of beam propagation, with the top of the skull horizontal (Fig. 5). During irradiation in lateral direction, the beam entered the head on the anatomical right side and exited on the left side. After lateral irradiation, the goniometer with the rat was rotated 90° clockwise, so that the centre of the beam array was now 3 mm to the right of the mid-sagittal plane for irradiation in anterior-posterior direction. The dose rate was continuously adjusted to 70 Gy/s by adjusting the exposure time to the storage ring decay.

In addition, we submitted eight tumour-free animals to MRT, in the same position as the tumour-bearing animals, three of which received an injection of BSO in the same localisation as the tumour-bearing animals. Five tumour-free animals served as healthy untreated controls.

After the radiotherapy, the animals were allowed to recover and the disease to run its course.

### Survival statistics

Due to the aggressive nature of the tumour, the change from being relatively well to sudden death was very fast and there was no need to euthanize animals according to our endpoint protocol. The survival curves are thus based on true survival data. Although the untreated animals that died before the scheduled therapy day are included in the graph of the survival curve, they are not included in the statistical analysis, which is based on 8 animals per experimental group. For data analysis, we used Kaplan-Meier curves. Logrank test (chi square statistic) was used to evaluate the *p*-values.

### ORT (assessment of new memory formation)

Cognitive dysfunction is frequently reported in patients after cerebral irradiation with conventional sources, especially in patients who were irradiated at a young age [36, 37]. In an earlier experiment, we had seen that the administration of BSO led to a significant temporary deficit in new memory formation [27]. The tumour-bearing animals in the current experiment did not survive long enough to conduct a meaningful assessment of the development of memory function. However, we were able to follow up the irradiated tumour-free animals which had served as controls, to assess the effect of MRT on memory function in the normal brain.

Rodents show a strong tendency to approach and explore novel rather than familiar objects. This feature of cognitive behaviour, related to the animals’ ability to form new memory contents, is exploited in the Object Recognition Test (ORT) developed by Ennaceur and Delacour [38].

For memory assessment, each animal was habituated to the empty test environment (a 40 cm × 40 cm × 60 cm open black Perspex box with a light grey floor) during a single session of 4 min duration. The following day, the animals were returned to the test environment where two identical objects had been placed on the floor of the cage (test part T1). Each animal was allowed to explore the environment including the two equal objects for 4 min before returning to its home cage. Confronted with this scenario, animals will typically spend equal times exploring each of the objects. The time spent exploring each of the objects was recorded for each animal. After the end of the test period, animals were returned to their home cages. Retrieved again from their home cages after a 4 h inter-trial interval, animals were inserted in the test environment for the second part of the ORT (test part T2). For this second session, one of the already familiar objects was replaced by a new object with similar salience. As before, the time spent exploring each of the objects was recorded for each animal. This test is based on the hypothesis that, in the second part of the test (T2), rats with normal memory function will spend more time examining the new rather than re-exploring the previously encountered object. Animals with memory encoding or retrieving impairments, however, will again spend equal times exploring both objects.

There is an ongoing debate regarding the relation between hippocampal function and performance in the new object recognition test. Results of a metaanalysis published in 2015 support the idea that the validity of the ORT depends on the length of interval between the two test sessions: the hippocampus appears to be necessary for object recognition memory only if the recall interval is longer than 10 min [39]. Thus, with a recall interval of 2 h between the test sessions in our study, the ORT is a valid tool for the assessment of object recognition memory. The ORT was conducted at 1 and 13 months after MRT.

## Results

### Gain of survival time

**Fig. 6:**
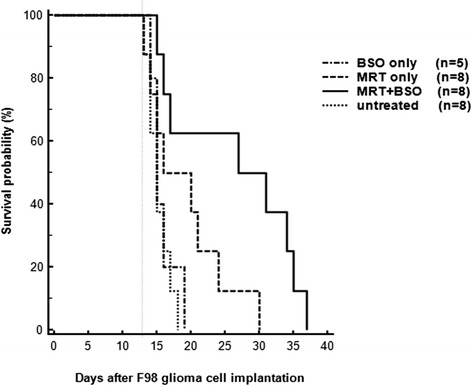
Survival probability of tumour-bearing animals. One half of the irradiated animals received an injection of the radioenhancer BSO into the tumour two hours prior to radiotherapy. The vertical dotted line marks the day of irradiation. MRT was conducted on Day 13 after tumour cell implantation. Eight animals were injected with BSO 2 h prior to irradiation

The increase of survival times within the irradiated groups, compared to untreated tumour-bearing animals, is illustrated in Fig. 6. Compared to MRT alone, we observed a significant additional gain in mean survival time when the glutathione synthesis inhibitor BSO was injected into the tumour 2 h prior to radiotherapy. The differences in mean survival times within treated groups were statistically significant between untreated tumour-bearing animals and rats in both irradiated groups: MRT only (*p* = 0.010) and MRT + BSO (*P* = 0.003).

All untreated tumour-bearing animals had died by day 20 after tumour cell implantation, with a mean survival time of 15 days (SD ± 1.85). Median survival was also 15 days, equal to the median survival seen in tumour-bearing animals in a previous experiment with identical tumour parameters who received BSO only. Median survival was 20 days for animals undergoing MRT only and 27 days for animals undergoing MRT + BSO injection prior to radiotherapy. The confidence interval for both the mean and the median was 95%.

Out of all other experimental groups, survival at day 20 after tumour cell implantation was 62.5% (5 animals) in Group A (MRT only) and 75% (6 animals) in Group B (MRT + BSO). On day 30 after tumour cell implantation, survival was 12.5% in Group A and 62.5% in Group B. There were no survivors on or beyond day 35 in Group A, but 25% (2 animals) were still alive in Group B. The last animal in Group B died 41 days after tumour cell implantation (Table 1). Thus, out of the groups containing tumour-bearing animals, the animals which underwent MRT with adjuvant BSO injection experienced the largest survival gain. Since the longest-surviving tumour-bearing animal in the untreated control group died 18 days after tumour cell implantation, this means that the survival time for 25% of animals in the MRT-BSO group had doubled.

**Table 1:** Survival rates in the experimental groups after tumour cell implantation

	No treatment	MRT	MRT + BSO
Day 13 Irradiation	8	8	8
Day 15	4	8	8
Day 20	0	5 62.5%	6 75%
Day 30	0	1 12.5%	5 62.5%
Day 35	0	0	2 25%
Day 40	0	0	1 12.5%

Survival rates are given as numbers of animals alive and as percentage of surviving animals in the experimental group (*n* = 8/group)

*MRT* microbeam radiation therapy, *BSO* Buthionine-SR-Sulfoximine, used as radioenhancer, injected into the tumour 2 h prior to radiotherapy

### MRT, BSO and new memory formation in tumour-free animals

**Fig. 7:**
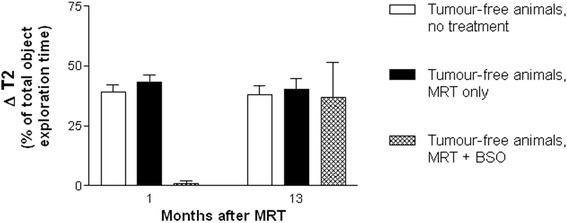
Memory function recorded in tumour-free animals at second exploration, at 1 and 13 months after MRT. MRT alone did not cause significant differences in non-irradiated animals. BSO caused a highly significant temporary inability for new memory formation

No significant memory deficit was detected either early or late after MRT alone (Fig. 7). This is in accordance with our previous results seen in a C6 glioma model [27]. As in this previous study, we observed that in the Fischer rats memory formation was also significantly impaired one month after MRT preceded by direct BSO injection into the brain. In our previous experiment, the object recall was on average very poor in healthy animals who received BSO injections before irradiation. Conversely, in the present experiment, we found a variability of memory performance between the three Fischer rats, with two of the three animals showing only little or no memory deficits while one animal showed no object recall at all. However, with only three animals in this group, this could have been purely by chance and this experiment should be repeated with larger animal numbers.

## Discussion

Survival times for ≥90% of untreated intracerebral tumour-bearing control animals were about 20 days or more in most small animal models previously used for radiotherapy studies at the synchrotron [21, 23, 40–43]. In our study, none of the animals survived beyond day 19 after tumour cell implantation. About 12% of animals died on or before day 13 after tumour cell implantation. Thus, they never reached the timeline set for the start of therapy. Thus, by implanting 100,000 F98 glioma cells, we used not only a small animal model of highly malignant brain tumour, but also an advanced stage of disease development. Nevertheless, we still were able to achieve a significant increase in survival time in two treatment groups using the MRT protocol.

Biston and colleagues, using an orthotopic F98 brain tumour model generated from 1000 implanted cells, reported a median survival time of 25 days for untreated tumour-bearing animals and a 31 day median survival after 5 Gy irradiation with synchrotron X-rays at 78.8 keV [41]. In our experiment, the number of injected cells was two orders of magnitudes higher and resulted in a median survival time of only 14 days. MRT alone resulted in a median survival time of 20 days. MRT following an injection of the glutathione synthesis inhibitor BSO added another 10 days to median survival time: One third of the overall survival time in this experimental group was contributed by the glutathione synthesis inhibitor BSO. Thus, we have extended the median survival time of animals bearing a malignant brain tumour in a very advanced stage beyond the median survival time seen in a much less advanced stage of the same tumour if untreated.

Biston et al. [41] showed that survival times after comparable irradiation doses administered as 6 MeV photons were equal or lower than those after synchrotron irradiation at 78.8 keV. Thus, by treating an F98 glioma at a very advanced stage of development with a combination of MRT and BSO, we have achieved a mean survival time similar to that achieved by 5 Gy with 6 MeV photons in a much less advanced F98 tumour.

Dose escalation by spatial hypofractionation for better tumour control is not entirely new in the history of radiotherapy for patients with glioblastoma multiforme. Stereotactic radiosurgery has been used successfully to boost tumour target doses beyond 60 Gy and increase overall survival [44, 45]. Following this line of thought, it would be an interesting approach to use MRT as integrated boost, with a valley dose equal to a dose administered in a single fraction of conventional radiotherapy. A survival gain might be achieved not only due to the high peak doses, but also from the bystander effects occurring in cells between the paths of the microbeams [46–49].

Few studies have investigated potential adverse effects of MRT on brain function. Earlier work has suggested that the structure of normal tissues in the path of the beam is greatly preserved after MRT [28, 50]. We have previously shown in an animal model of a less advanced malignant brain tumour and in healthy control animals that, while BSO injections caused significant deficits in new memory formation, those deficits were temporary in tumour-bearing animals. MRT alone did not cause significant deficits in the formation of new memory [27]. This observation supports the concept of administering whole brain irradiation of the MRT type at advanced tumour stages, when tumour control cannot be achieved by focussed, locally limited irradiation alone.

F98, like the human glioblastoma multiforme that it is supposed to model, is characterized by extensive invasion of normal brain structures with tumour cell clusters located at varying distances from the primary tumour bulk, also along the Virchow-Robin spaces [26, 51–53]. Thus, a considerable number of tumour cells could have been outside the field of irradiation in our study. This might be one of the explanations why, contrary to the C6 glioma model, we saw no long-term survivors in F98-bearing animals in our experiment. As no negative influence of MRT alone was seen on new memory function in tumour-free animals, the integration of MRT into a whole brain irradiation concept seems reasonable.

Interestingly, it has been found that after intravenous injection the rate of BSO entry into gliomas is higher than the entry into tumour-free brain, by about one order of magnitude [54]. Further, the injection of BSO into the tumours caused small haemorrhages in about 50% of the animals in our experiment. Therefore, one might consider exploring different routes of BSO administration to avoid an invasive procedure and the risk of inducing a potentially fatal haemorrhage in highly vascularized tumours. An efficient reduction of tumour burden has, for instance, been shown in an animal model of oesophageal cancer, where BSO was dissolved in drinking water at a concentration of 20 mM [55]. In an orthotopic glioma model, a reduction of tumour glutathione to about 8% of the non-treated control values was achieved by a combination of intraperitoneal and oral BSO administration [14].

The adjuvant administration of BSO significantly increases survival times in several types of malignant brain tumour in vivo [24, 56]. The memory deficits caused by the local injection of BSO into C6 glioma prior to radiotherapy were temporary [27]. Thus, in a clinical situation, patients might choose a gain of survival time despite of temporary memory deficits, especially when the gain is expected to last several times longer than the period of memory deficits.

Data obtained in a recent pre-clinical study support the concept of an intravenous administration of BSO as radioenhancer [57]. Those data demonstrated that the increase in vascular permeability caused by MRT was significantly higher in the tumour-supplying vasculature than in mature blood vessels in normal tissue.

The results of at least two Phase I clinical trials in which BSO was administered intravenously as adjuvant therapy in patients with solid malignant tumours have been published [55, 56]. An intracellular GSH depletion to about 30–40% of the baseline levels was induced, resulting in a significant inhibition of y-glutamylcysteine synthetase, the rate-limiting enzyme of GSH synthesis [58]. This transient inhibition of y-glutamylcysteine synthetase declined gradually within 6–12 h after the end of the BSO infusion. The only reported adverse effect of BSO administration was occasional nausea.

Furthermore it was shown in vitro and in vivo that BSO increased the sensitivity to temozolomide, the standard chemotherapy agent in the treatment of patients with high grade glioma, by modifying ROS production [59, 60]. Based on these data, BSO could be administered to patients with high grade glioma to increase the sensitivity of their tumour cells to temozolomide. The expectation would be that BSO induces oxidative stress by depleting intracellular glutathione levels, subsequently decreasing the anti-oxidative reserves of the cancer cells and thus inducing apoptosis.

The preliminary results obtained in our pilot experiment should be confirmed in an expanded study with pre-therapeutic imaging to ensure that all tumours were of similar size at the time of treatment and to include a group of tumour-bearing animals that just receives an injection of BSO and no radiotherapy. Based on all data, the integration of MRT into a clinical schedule of whole brain irradiation could then be tested, possibly in combination with an intravenous administration of the glutathione synthesis inhibitor BSO. This might contribute to a significantly better tumour control even in a brain tumour at a very advanced stage of the disease.

Furthermore, it would also be worthwhile to conduct a well-designed experiment using BSO as adjuvant with broad beam irradiation, with and without MRT as integrated boost. Such an experiment might answer the question whether even with conventional radiotherapy, patients might benefit from adjuvant BSO.

## Conclusions

The results of this pilot study suggest that MRT in combination with the glutathione synthesis inhibitor BSO results in a significant increase of mean survival time in an orthotopic small animal model of highly malignant brain tumour in a very advanced stage of development. Survival time was doubled compared to untreated animals. Two thirds of the time gained can be attributed to the glutathione synthesis inhibitor. A follow-up study in a larger number of animals is required to increase statistical power and confirm these promising results.

We have also shown that MRT alone does not impair new memory formation. These preliminary results should be confirmed in an expanded study.

## Abbreviations

BSO: Buthionine-SR-Sulfoximine
ESRF: European Synchrotron Radiation Facility
MRT: Microbeam radiation therapy
ORT: Object recognition test
PVDR: Peak-to-valley dose ratio
